# Work- and mental health-related events and body mass index trajectories during the Covid-19 lockdown. Evidence from the lifelines cohort study in the Netherlands

**DOI:** 10.1038/s41366-023-01421-2

**Published:** 2023-12-02

**Authors:** Lluís Mangot-Sala, Nynke Smidt, Aart C. Liefbroer

**Affiliations:** 1 Netherlands Interdisciplinary Demographic Institute (NIDI)—Royal Netherlands Academy of Sciences (KNAW), The Hague, the Netherlands; 2 Department of Epidemiology, University Medical Center Groningen (UMCG), University of Groningen (RUG), Groningen, the Netherlands; 3 Department of Sociology, Vrije Universiteit Amsterdam (VU), Amsterdam, the Netherlands

**Keywords:** Risk factors, Epidemiology

## Abstract

**Background:**

The aim of this study was to identify heterogeneity in trajectories of body mass index (BMI) during the Covid-19 pandemic in the Netherlands. Moreover, we aimed to investigate whether work- and mental health-related disruptive events experienced during the pandemic, such as job insecurity or depression, were associated with such BMI trajectories.

**Methods:**

Longitudinal data from the Lifelines Covid Questionnaire was used (21 waves between April 2020 and July 2021; *n* = 64,630). Different trajectories were identified using group-based trajectory models. Multinomial regression models were fitted to analyse the main determinants of experiencing changes in BMI during the pandemic.

**Results:**

Trajectories of increased BMI, and, to a lesser extent also trajectories of decreased BMI, were more common among those who experienced disruptive work-related events (e.g., being laid-off or having a temporary contract) and mental health-related events (e.g., anxiety or depression) during the pandemic. Those experiencing multiple events were particularly likely to show trajectories of increased or decreased BMI.

**Conclusions:**

During the Covid-19 pandemic, strong heterogeneity was observed in BMI trajectories. This was partially related to work- and mental health-related events.

## Introduction

Overweight and obesity are a growing public health concern. Despite being mainly caused by preventable factors, the prevalence of overweight has tripled in the last 50 years and, nowadays, affects around 60% of adults in Europe [1]. Obesity, often defined as having a body mass index (BMI)—i.e., a person’s weight divided by their height and expressed in kg/m2- of 30 or higher, is a major cause of morbidity and mortality [2] and an important risk factor for chronic conditions, such as cardiovascular diseases, diabetes, dementia [3] and multiple cancers [4, 5]. Moreover, obesity has been reported to be associated with higher risk of severe Covid-19 outcomes, including hospital admission and mortality [1, 4, 5], which, in turn, increased stigmatisation of people with obesity during the pandemic [6].

Obesity is the result of a complex interaction of environmental, biological, psychological, social and behavioural factors [1, 7]. While health behaviours, such as diet or physical activity, clearly are associated with it [8], there is robust evidence that it is also associated with individuals’ socio-economic conditions [9]. Moreover, disruptive events -also known as stressful events or stressors- [10] have been shown to be related to obesity as well [11–14].

Indeed, disruptive events, and events that affect an individual’s sense of identity [10] and locus of control [9] in particular, are known to have a wide range of negative health consequences. A widely accepted explanation is that the stress caused by these events leads to biological changes in the body [7, 13] via the release of hormones, such as cortisol, adrenaline and noradrenaline [9], and/or inflammatory processes [15]. Consistently, evidence suggests that stress associated with disruptive events can lead to changes in BMI [13].

The COVID-19 pandemic and its associated lockdowns—i.e., all preventive measures implemented to control the spread of the virus-, is a collective disruptive event that could lead to a large amount of uncertainty about the extent and duration of the pandemic [16], financial insecurity [17] and, consequently, to an overall increase of stress levels [17, 18]. Moreover, social interactions that otherwise may buffer the negative impact of disruptive events [19], were significantly reduced [17], limiting potential coping mechanisms. Given that stress can potentially lead to both weight gain and weight loss [20], a first aim of this study is to examine BMI trajectories during the COVID-19 pandemic.

A second aim of the study is to examine which “individual” disruptive events experienced during the pandemic were related to weight changes. The pandemic affected multiple life domains. First, the *work domain* was affected. Many individuals were laid-off due to business closure [21], which increased financial insecurity [22, 23]. Among those who kept their jobs, many had to suddenly work from home, with the ensuing adjustment. Findings are mixed, with some studies reporting an overall positive effect of working from home, due to greater perceived work control and better work-life balance [24], whereas others showed increased stress levels [17]. Yet another relevant group were those having “essential jobs” during the COVID-19 outbreak—e.g., in health-care, retail, food processing or education^1^-, who faced a higher risk of viral infection, as well as higher stress levels [18, 25].

Second, the pandemic was a serious threat to *health*, which raised fear, particularly for those at higher risk of severe Covid-19 outcomes, such as older individuals, and those with pre-existing chronic conditions, or obesity [4]. Consistently, studies have shown that stress levels during the first months of the pandemic were particularly high among patients with chronic diseases [18]. Moreover, the pandemic seemed to reinforce pre-existing mental disorders, as individuals reporting pre-existing depression or anxiety disorders experienced a larger increase in their symptoms [17].

Disruptive events do not occur in isolation but are often intertwined [11]. Moreover, their negative effects tend to accumulate, often showing a dose-response pattern [26], as reported by studies testing the impact of disruptive events on mental health [27] and alcohol abuse [28]. Evidence is particularly rich regarding the cumulative impact of disruptive events experienced during early life -considered a “sensitive period” [29]- on BMI and obesity in later life [11–13]. Yet, the short-term impact of simultaneous events occurring during adulthood remains largely unexplored.

Several studies have analysed the impact of the Covid-19 lockdown on BMI [2, 30–32]. However, changes in BMI were either assessed retrospectively [30, 31], or relying on a small number of observations [2, 32, 33]. Yet, longitudinal trajectories of BMI should be analysed in order to properly identify the determinants of obesity [34]. Examining *changes* in BMI is important, as evidence has shown that a stable BMI is associated with lower mortality rates [35] and better health [36], whereas changes in BMI are associated with increased mortality [34, 36]. Furthermore, examining BMI trajectories may help unravel heterogeneity, which in turn could help define the key determinants of weight change.

Only a few studies have addressed heterogeneity in changes in BMI during the pandemic: a systematic review and meta-analysis reported weight gain among a large group of individuals (11.1–72.4%) and weight loss among a somewhat smaller group of older adults (>60 years old) (7.2–51.4%) after the first lockdown period [33]. However, only one of the 36 studies included in the analysis was longitudinal [2], and no study considered the role of disruptive events experienced during the pandemic.

All in all, there is evidence that the pandemic triggered a series of disruptive events, which increased stress levels for many individuals, but their relation to BMI changes during the pandemic is unclear. This longitudinal study improves upon previous studies that rely on a pre-post assessment, have fewer waves of data and/or small sample sizes. We rely on a large sample (*n* = 64,630) and 21 waves of panel data gathered over a period of 15 months, covering an extensive part of the COVID-19 pandemic. We aim to: (1) analyse BMI trajectories during the Covid-19 pandemic; (2) identify the main determinants of experiencing meaningful changes in BMI during the pandemic.

## Methods

The Lifelines COVID-19 Questionnaire was launched within the Lifelines Cohort Study, a multi-disciplinary prospective population-based cohort study examining in a three-generation design the health and health-related behaviours of 167,729 persons living in the northern Netherlands. It employs a broad range of investigative procedures in assessing the biomedical, socio-demographic, behavioural, physical and psychological factors which contribute to the health and disease of the general population, with a special focus on multi-morbidity and complex genetics -composition and characteristics of the sample have been discussed elsewhere [37].

The Lifelines COVID-19 Questionnaire assesses the effects of the pandemic, attitudes towards the COVID-19 regulations, and health (behaviours) during the pandemic [38]. In 24 waves, participants were asked to fill out detailed web-based questionnaires. For the purpose of our study, 21 waves of the COVID-19 questionnaire were used -waves 10, 16 and 18 did not contain information on body weight-, covering the period between April 2020 and July 2021. A total of 76,795 participants from the Lifelines Cohort Study participated in the COVID-19 cohort. Individuals with missing values in body weight (*n* = 3980) and those with only one observation (*n* = 10,825) were dropped from the analyses, leading to a final sample of 761,062 observations nested in 64,630 individuals (60.1% female; mean age 55.1 years), with 11.8 observations/person on average. The Lifelines protocol was approved by the UMCG Medical ethical committee, and all participants signed an informed consent form.

### Measurements

#### Outcome variable

Body Mass Index (BMI) was calculated as weight (in kilograms) divided by the square of height (in metres). Self-reported weight was assessed in all waves, whereas height was measured objectively by health-care professionals in “Assessment 2B” of the Lifelines Cohort Study. Extreme values were trimmed according to WHO guidelines [1] (BMI < 15 was given the value “15” and BMI > 45 the value “45”). Next, our outcome of interest “*Changes in BMI*” was calculated, subtracting BMI at baseline from the BMI value in each subsequent observation. A robustness check with changes in BMI in percentage points was carried out, leading to nearly identical results.

#### Exposure variable

A time variable “Days since first lockdown (15th of March, 2020)” was created and used as independent variable in the group-based trajectory analyses in order to analyse changes in BMI over time.

#### Covariates

Two sets of covariates for the work and health life domains were created. First, variables regarding the work domain contained an assessment of *employment status at baseline* (“employed” -including full- and part-time- as reference, “retirement”, “unemployment”, “occupational disability”, -defined as “receiving disability benefits due to long-term illness”, based on the Dutch classification system-, and “others”, containing students, homemakers, maternity leave, etc.), as well as *work-related disruptive events* experienced during the pandemic. The latter were given value “1” if the event was reported at least once during the observation period and contained the following events: *having an essential job*, as defined by the Dutch government [38]; having been *laid-off due to the pandemic* (including paid and unpaid leaves, and forced sick leave); *working from home*; *working as a freelancer*; and having a *temporary contract* during the pandemic.

Second, the health domain contained the following assessments: health status at baseline, including having a *chronic disease at baseline* (based on the question “Do you have a chronic health condition? (yes/no)”); and *BMI at baseline*, assessed categorically: “Healthy BMI (<25)”, “Overweight (25–29.9)” and “Obesity (≥30)” [1] -the few individuals reporting “underweight (<18.5) (*n* = 326; 0.51%) were included in the “Healthy BMI” category-. In turn, the following *mental health-related events* were assessed: experiencing *feelings of loneliness* (based on the question “how socially isolated have you felt in the last 7 days?” (14 days from wave 6 onwards); values ranged from 1 (“no social isolation”) to 10 (“extreme isolation”)). The variable was dichotomised to facilitate the interpretation (score ≥7 considered threshold for “loneliness”, based on previous studies [39]). *Depression* and *anxiety symptoms* were assessed through the Mini International Neuropsychiatric Interview (MINI). Based on DSM-5 criteria, depressive disorder was defined as reporting “≥5 depressive symptoms, among which depressive mood or loss of interest in every-day activities”; anxiety disorder as “reporting ≥3 anxiety symptoms, “excessive worry/apprehensive expectation that the individual finds difficult to control” among them [40, 41].

Third, a variable with the total number of work- and mental health-related stressful events that participants experienced was created, values ranging from “No events” to “5+ events”. Last, the following sociodemographic covariates were included in the models: *Living arrangements* (living with partner/family, and living on their own; additionally, *having children (<18 years old)* was assessed through questions regarding the ages of the cohabitants). *Sex* (male/female); *Age* at baseline was categorised in age groups (<40, 41–50, 51–60, 61–70 and >70); and *Educational Level* was based on categories of the Dutch educational system: “low” (up to general secondary education), “middle” (secondary vocational education/higher pre-university education), and “high” (higher professional education/university education).

### Statistical analyses

First, group-based trajectory models (GBTM) with the outcome “Changes in BMI” and the exposure time variable were estimated. GBTM are a form of finite mixture models, assuming multiple groups within the population with different trajectories over time [42]. These models included polynomial terms and controlled for BMI at baseline. The selection of the number of groups was based on two steps: first, different solutions of the GBTM, from two to seven groups, were compared using Akaike and Bayesian information criterion values (see Table A1 in Appendix 1). Yet, the accuracy of these values has been shown to be highly dependent on data features (e.g., sample and class size) [43] and to favour models with an unlikely high number of groups [13]. Hence, we followed Nagin’s guidelines for model selection [42], namely: (a) close similarity between the probability of group membership and the proportion assigned to that group (posterior probability of group membership); (b) odds of correct classification (OCC) for each group exceeding a minimum threshold of 5; (c) average posterior probabilities (APP) for each group exceeding 0.7; and (d) each group comprising >5% of the sample. Based on these criteria, the best solution was selected. Finally, groups were labelled accordingly and coded as a categorical variable.

Second, in order to analyse the main determinants of each BMI trajectory, multinomial logistic regression models were fitted, including baseline characteristics and work- and mental health-related events experienced during the pandemic. Based on these models, marginal effects were estimated. Due to the high number of tests performed, which could increase the risk of finding spurious associations, the significance level was adjusted to <0.01.

Last, a set of sensitivity analyses was performed: models were stratified by sex (Appendix 2), and potential attrition bias was assessed (see Appendix 3), including a model with imputed data (Table S6), which showed very similar coefficients to the one presented below. All analyses were run with Stata 13.1.

## Results

The main characteristics of the study sample (*n* = 64,630) are described in Table 1. Most participants were women (60.99%) and the average age was 55 years old. Most individuals were employed (64.49%), whereas a large group (21.91%) was retired. About one third of the sample reported working exclusively from home for at least some time during the pandemic, and 29.85% had a so-called “essential” job. About a quarter of the sample (26.32%) had a chronic disease at baseline. While 42.46% had a healthy BMI, a similar group in size reported overweight, and 16.33% had obesity at baseline. Most individuals (54.56%) experienced loneliness at some point, whereas anxiety and depression were less common, albeit still affecting 13.20% and 6.47% of the population.

**Table 1:** Characteristics of the sample at first observation (*n* = 64,630).

	*n* (%)	missing (%)
Work status at baseline		4 (0.01%)
Employed	41,677 (64.49%)	
Retired	14,162 (21.91%)	
Unemployed	1795 (2.78%)	
Occupationally disabled	1600 (2.48%)	
Other	5392 (8.34%)	
Work-related events^a^		2 (0.00%)
“Essential” Job	19,294 (29.85%)	
Laid-off due to covid	4675 (7.23%)	
Work from home	21,076 (32.61%)	
Freelancer	6551 (10.14%)	
Temporary contract	3982 (6.16%)	
Nº of work-related events		2 (0.00%)
0	27,004 (41.78%)	
1	22,552 (34.89%)	
2	12,389 (19.17%)	
3+	2683 (4.15%)	
Health status at baseline		
Body Mass Index (BMI)		0 (0.00%)
Healthy	27,468 (42.46%)	
Overweight	26,628 (41.20%)	
Obesity	10,535 (16.33%)	
Chronic disease	17,009 (26.32%)	8 795 (13.61%)
Health-related events^a^		
Loneliness	35,265 (54.56%)	221 (0.34%)
Depression	4183 (6.47%)	8 (0.01%)
Anxiety	8533 (13.20%)	7 (0.01%)
Nº of health-related events		228 (0.35%)
0	26,706 (41.78%)	
1	30,012 (46.44%)	
2	5112 (7.91%)	
3	2572 (3.98%)	
Total nº of events		230 (0.36%)
0	10,820 (16.74%)	
1	23,047 (35.66%)	
2	16,792 (25.98%)	
3	9444 (14.61%)	
4	3196 (4.95%)	
5+	1101 (1.70%)	
Living arrangement		2 (0.00%)
With partner/family (only adults)	41,384 (64.03%)	
With partner/family (& children)	16,511 (25.55%)	
Living alone	6733 (10.42%)	
Age (mean; SD)^b^	55.10 (12.04%)	0 (0.00 %)
Age group		0 (0.00 %)
≤40 years	8304 (12.85%)	
41–50 years	12,943 (20.03%)	
51–60 years	23,258 (35.99%)	
61–70 years	12,886 (19.94%)	
>70 years	7239 (11.20%)	
Sex		0 (0.00 %)
Male	25,215 (39.01%)	
Female	39,415 (60.99%)	
Educational Attainment		675 (1.04%)
Low	16,691 (25.83%)	
Middle	24,951 (38.61%)	
High	22,313 (34.52%)	

^a^Events reported at least once during the pandemic

^b^Average of the whole sample.

### BMI trajectories

**Fig. 1:**
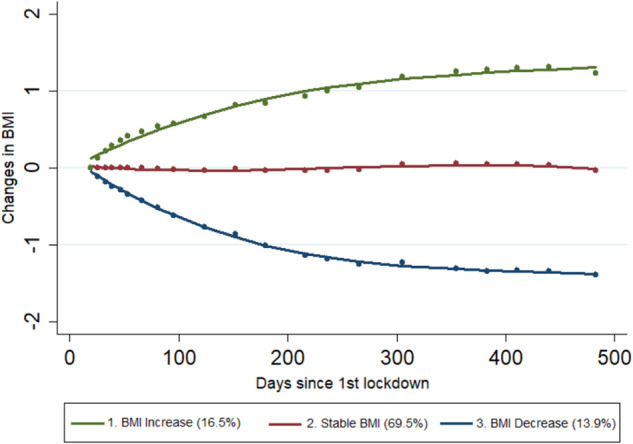
Group-based trajectory models of changes in BMI during the Covid-19 pandemic. Three-group solution.

Next, group-based trajectory models were performed, and a three-group solution was chosen. The model (Fig. 1 and Table 2) showed one large group of individuals (69.5% of the sample) who did not show major changes in their BMI (“Stable BMI trajectory”), another group showing a substantial BMI increase (“BMI increase trajectory”; 16.5%) -with an increase of over 1 BMI unit on average-, and a third group showing a substantial decrease in BMI (“BMI decrease trajectory”; 13.9%), with a decrease of over 1 BMI unit on average. Other models (e.g., a 5-group solution) would identify smaller groups with even stronger fluctuations in BMI (±2 BMI units on average), as shown in Table S1 & Fig. S1 in Appendix 1. Yet, the 3-group solution was the most parsimonious, and had a better fit in statistical terms, based on Nagin’s guidelines [39]. Moreover, sensitivity analyses comparing the most “extreme” groups of the 5-group and the 3-group solution did not alter our main conclusions (Fig. S2 in Appendix 2).

**Table 2:** Factor estimates for the three-group trajectory solution.

Trajectory	*n* (%)	A.P.P^a^	O.C.C.^b^	P.P.^c^
1 “BMI increase”	9253 (14.32%)	0.94	81.11	16.19
2 “Stable BMI”	47,574 (73.61%)	0.92	5.70	68.11
3 “BMI decrease”	7803 (14.32%)	0.94	106.86	13.66

^a^Average posterior probabilities;

^b^Odds of correct classification;

^c^Posterior probabilities.

### Determinants of each BMI trajectory group

Table 3 shows the determinants for falling into each of these groups, taking group #2 (“Stable BMI trajectory”) as reference. Moreover, we stratified these models by sex as a sensitivity analysis (Fig. A3 in Appendix 2). We now discuss the main groups of determinants of experiencing meaningful changes in BMI (i.e., falling into trajectory group #1 or #3):

**Table 3:** Determinants of BMI trajectory group. Multinomial regression models (*n* = 54,521).

Ref. Group #2. Stable BMI	Group #1 BMI Increase		Group #3 BMI Decrease	
	OR	CI 95%	OR	CI 95%
Work status (Employed)	1		1	
Retired	0.85*	0.75–96	1.20*	1.07–36
Unemployed	1.03	0.88–1.19	1.38*	1.18–61
Occupationally Disabled	1.16	1.00–34	1.37*	1.17–46
Work-related events				
Essential job	1.09*	1.02–15	1.02	0.95–1.09
Laid-off due to COVID	1.17*	1.07–28	1.05	0.94–1.16
Work from home	1.10*	1.03–17	1.13*	1.05–1.21
Freelancer	1.02	0.93–1.11	1.09	1.00–1.20
Temporary job	1.18*	1.07–1.30	1.26*	1.13–1.40
Chronic health condition	1.20*	1.14–1.27	1.06	1.00–1.12
BMI at Baseline (“Healthy weight”)	1		1	
Overweight	1.65*	1.56–1.75	2.22*	2.08–2.37
Obesity	2.46*	2.30–2.64	4.23*	3.93–4.56
Health-related events				
Loneliness	1.36*	1.29–1.43	1.12*	1.06–1.18
Depression	1.41*	1.28–1.56	1.29*	1.15–1.44
Anxiety	1.20*	1.11–1.29	1.20*	1.10–1.31
Living arrangement (Family, no children)	1		1	
Family, with children	1.01	0.95–1.08	0.95	0.89–1.03
Lives alone	1.17*	1.08–1.28	1.02	0.93–1.11
Age (51–60 years)	1		1	
<=40 years	1.45*	1.34–1.57	1.03	0.94–1.13
41–50 years	1.14*	1.06–1.22	0.91	0.83–0.99
61–70 years	0.88*	0.80–0.96	1.02	0.92–1.11
>70 years	0.87	0.76–1.01	1.07	0.94–1.23
Sex (Male)	1		1	
Female	1.50*	1.42–1.59	0.99	0.94–1.05
Educational Attainment (High)	1		1	
Middle	1.06	1.00–1.13	0.94	0.88–1.01
Low	1.10*	1.03–1.18	0.88*	0.82–0.95

*p < 0.01.

#### Work domain

As shown in Table 3, work status at baseline was associated with changes in BMI during the observation period. Thus, those who were unemployed (OR = 1.38), occupationally disabled (OR = 1.37) or retired (OR = 1.20) at baseline were more likely to experience weight loss than employed individuals. In turn, work-related events experienced during the pandemic were associated with changes in BMI: having a temporary job during the pandemic was the strongest work-related determinant of both increased (OR = 1.18), as well as decreased (OR = 1.26) BMI trajectories. Working from home was also associated with changes in both directions, albeit the association was somewhat weaker (OR = 1.10 and OR = 1.13, respectively). Last, those who were laid-off due to the Covid-19 outbreak (OR = 1.17) and, to a lesser degree, those with an “essential job” (OR = 1.09) were at higher risk for being in the BMI increase trajectory.

#### Health domain

Health status at baseline also was associated with weight changes. Individuals who reported having a chronic disease were overrepresented in the BMI increase trajectory (OR = 1.20), even accounting for mental health symptoms. Moreover, higher baseline BMI levels were associated with experiencing changes in both directions, particularly weight loss. In turn, those who experienced mental health symptoms during the pandemic were significantly more likely to be in both the BMI increase, as well as the BMI decrease trajectory, the association being particularly strong for depression symptoms (OR = 1.41 and OR = 1.29, respectively), followed by loneliness (OR = 1.36 and OR = 1.12) and anxiety (OR = 1.20 for both trajectories).

#### Cumulative effects

**Fig. 2:**
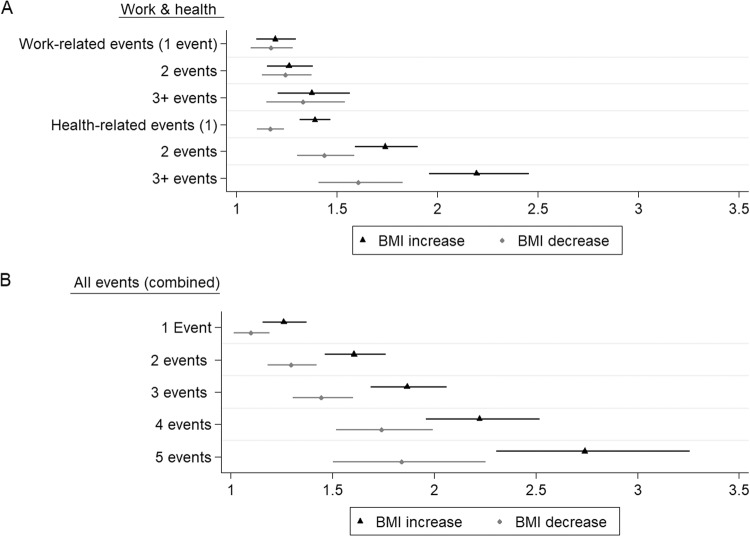
Cumulative effects of work- and mental-health related events on predicting increased and decreased BMI during the COVID-19 lockdown (odds ratios). **A** Shows cumulative effects of work- and mental health-related events; **B** shows joint cumulative effects of all disruptive events.

Analyses with the number of events that participants experienced in the work- and mental-health domains show that the effects of such events accumulated in a dose-response pattern, particularly in the case of mental health-related events. As shown in Fig. 2 (and Tables S2 and S3 in Appendix 2), experiencing three mental health symptoms strongly increased the risk of weight changes, particularly weight gain (OR = 2.19), whereas those who experienced five or more events in total (*n* = 1147) reported the highest risk of being in either the BMI increase trajectory (OR = 2.74) or the BMI decrease trajectory (OR = 1.84). In both instances, the risk of having an increasing BMI-trajectory is larger than the risk of having a decreasing BMI-trajectory.

#### Sociodemographic characteristics

Last, sociodemographic characteristics were associated with BMI trajectories. Thus, those living on their own were overrepresented in the upward trajectory (OR = 1.17), compared with those living in a shared household with partner and/or family. However, having children (<18 years) at home did not seem to have an effect. Finally, women (OR = 1.50) and those with lower education (OR = 1.10) reported a higher risk of increased BMI, whereas age appeared as a clear protective factor against weight gain (OR = 0.88 for those >60 years old). The same models stratified by sex showed only a few significant differences (see Fig. S3 in Appendix 2).

## Discussion

This study contributes to the literature by showing that BMI trajectories during the pandemic were associated with work- and mental health-related factors. While most of the existing literature relied on a pre-post pandemic comparison, or at best a small number of observations, this study relied on a large sample of nearly 65,000 individuals followed during 21 waves of data, covering an observation period of 1.5 years. Results showed that, while a large part of the sample did not experience meaningful changes in their body weight, about 16% experienced a substantial increase in their BMI, and around 14% had considerable weight loss during the pandemic, in line with the meta-analysis by ref. [33].

Our study showed that several work-related events, such as having been laid-off or having a temporary contract during the pandemic, were associated with changes in BMI, both weight gain and weight loss. Previous studies had shown that individuals facing job precariousness reported higher perceived insecurity [23] and stress [44], which in turn led to negative health outcomes [23]. However, to our knowledge, no study had shown job insecurity to be associated with changes in BMI. Our interpretation would be that the occurrence of these events led to stress that could trigger not just unhealthy coping mechanisms (e.g., alcohol consumption [45] or binge eating [46]), but also biological changes in the body, e.g., altering body weight [13].

Consistently, we found mental health-related events, such as loneliness, anxiety or depression, to be strong determinants of changes in BMI, the latter increasing the odds of weight gain by 41% and the odds of weight loss by 29% [13]. Furthermore, individuals with overweight or obesity at baseline were more likely to experience body weight changes. This corroborates recent findings from a retrospective study showing that individuals with obesity faced more depression and anxiety symptoms, as well as stronger weight gain during the pandemic [47]. Thus, our longitudinal study advances on such cross-sectional evidence by showing that, even accounting for BMI at baseline, individuals who suffered from mental health issues during the pandemic were at increased odds to experience significant weight gain.

Furthermore, our study showed that disruptive/stressful events in the work and health domains are *independently* associated with the outcome. Moreover, findings show a clear dose-response pattern: the more health- and work-related events, the higher the risk for weight changes, particularly for increased BMI. This is consistent with the concept of “accumulation of risks” coined by life-course scholars [29], previously reported in the impact of adverse life events on BMI [48]. Our study is the first to show similar results regarding disruptive events experienced during the pandemic.

Last, our results suggested that certain groups of individuals, i.e., women, younger individuals, those with lower education, and those living alone were more likely to experience strong increases in their BMI than their counterparts, even accounting for work- and mental health-related factors. Although the mediating pathways may slightly differ across subgroups, previous studies showed that these groups reported both worse mental health [49], as well as higher difficulties to “control their BMI” [33] during the pandemic, which again suggests the importance of higher stress levels (and fewer alternative coping mechanisms [50]).

Our findings showed that some determinants were associated with both weight gain and weight loss. While this is a common pattern in trajectory-based studies [35, 46, 49], it raises several questions, e.g., why is feeling depressed for some individuals related to weight gain, but to weight loss for others? Although research has traditionally focused on risk factors of increased BMI [1, 12, 30, 46], there is evidence that losing weight can also be indicative of high stress and other underlying conditions [33]. In fact, changes in appetite are reported as one of the core symptoms of depression [46], which could explain why, in our study, weight loss was more common among those reporting depression and anxiety. On the other hand, weight loss can also be due to a healthier lifestyle, such as healthier diet or increased physical activity, reported during the first lockdown [30]. However, a longitudinal study showed contrasting results: while some individuals increased their food intake, others—i.e., women, single/divorced individuals and those having depression or anxiety symptoms- declared eating less as a reaction to the pandemic [46]. Future research should disentangle this conundrum by analysing the mediating role of health behaviours.

This study has several limitations. First, we did not have information on ethnicity of the participants. Therefore, the ethnic composition of our sample may be slightly different from the Dutch population. Moreover, women, and older individuals are slightly overrepresented, whereas younger individuals are underrepresented, which could bias results. However, an analysis using multiple imputation using chained equation models (MICE)—see Table S6 in Appendix 3- provided results that were practically identical to those presented, suggesting little attrition bias. Second, there may be collinearity between some variables: e.g., having a chronic disease, occupational disability, depression and anxiety. However, stepwise deletion of these variables led to similar results, and since removing or combining them would imply a loss of information, they were kept in the models. Third, self-reported weight is often underreported [2]. Although using change score as outcome variable may diminish measurement error within individuals [2], participants may still underestimate changes in their body weight and inaccurately report the same weight across observations. Thus, changes reported in this study may underestimate the real changes in the study population. Last, although this study provides theoretical support for the mediating role of stress, stress levels could not be assessed due to data limitations. Further research could combine different stress measurements (e.g., self-reported stress as well as cortisol levels), in line with studies adopting a “multi-layered” approach, i.e., using different indicators, from health outcomes to biological markers [51], to test the “stress hypothesis”.

In sum, this study relied on a very large sample and numerous observations covering most of the pandemic, showing that systematic changes in BMI during the lockdown were associated with work- and mental health-related disruptive events experienced during the pandemic. Results are consistent with the idea that such stressful events contributed to changes in BMI. This has several implications: first, research should also examine the distal stressors associated with obesity, rather than only studying health behaviours, which, in fact, could be acting as mediators in the association between stress and BMI changes [7]. Second, the association between stressful events and BMI changes suggests that policies aimed to prevent overweight/obesity should try to prevent or buffer stressful living conditions, next to paying attention to lifestyle factors.

### Supplementary information

Supplementary Materials

## Data Availability

The datasets generated and analysed during the current study were provided by Lifelines under licence. Access to the data can be granted under licence by Lifelines. The codes used to produce the results presented in this paper are shared on: https://github.com/LluisMontag/BMI-Trajectories.

## Footnotes

1 https://www.cbs.nl/en-gb/faq/corona/economie/how-many-people-are-key-workers-in-the-netherlands-
