# Supplementary material for: Work- and mental health-related events and body mass index trajectories during the Covid-19 lockdown. Evidence from the lifelines cohort study in the Netherlands
Source: Int J Obes (Lond). 2023 Dec 2;48(3):346–52. doi: 10.1038/s41366-023-01421-2 (PMC10896728; doi:10.1038/s41366-023-01421-2)
Supplement: Supplementary file 1 — Supplementary Materials [file 41366_2023_1421_MOESM1_ESM.docx]

**Appendix 1. Sensitivity Analyses: Comparison of different trajectory solutions**

**Table S1. Statistics of the different group-based trajectory models**

| Nº of trajectories | BIC | Trajectory # | n | A.P.P^1^ | O.C.C.^2^ | P.P.^3^ |
| --- | --- | --- | --- | --- | --- | --- |
| 2-group | -616 803.70 | 1 | 15 122 | 0.93 | 36.06 | 27.32 |
|  |  | 2 | 49 508 | 0.92 | 4.80 | 70.64 |
|  |  |  |  |  |  |  |
| 3-group | -517 051.08 | 1 | 7 803 | 0.94 | 106.86 | 13.66 |
|  |  | 2 | 47 574 | 0.92 | 5.70 | 68.11 |
|  |  | 3 | 9 253 | 0.94 | 81.11 | 16.19 |
|  |  |  |  |  |  |  |
| 4-group | -478 649.97 | 1 | 3 828 | 0.93 | 203.96 | 6.33 |
|  |  | 2 | 13 269 | 0.90 | 26.94 | 24.95 |
|  |  | 3 | 40 370 | 0.85 | 4.81 | 54.51 |
|  |  | 4 | 7 163 | 0.94 | 112.96 | 12.18 |
|  |  |  |  |  |  |  |
| 5 group | -452 215.31 | 1 | 2 956 | 0.93 | 268.04 | 4.77 |
|  |  | 2 | 8 463 | 0.91 | 50.92 | 16.01 |
|  |  | 3 | 38 189 | 0.82 | 4.49 | 50.04 |
|  |  | 4 | 11 220 | 0.89 | 29.23 | 21.08 |
|  |  | 5 | 3 802 | 0.93 | 204.78 | 6.07 |
|  |  |  |  |  |  |  |
| 6 group | -433 309.22 | 1 | 2 245 | 0.93 | 385.73 | 3.55 |
|  |  | 2 | 2 704 | 0.91 | 166.17 | 5.51 |
|  |  | 3 | 9 419 | 0.88 | 34.85 | 16.89 |
|  |  | 4 | 37 347 | 0.81 | 4.57 | 48.59 |
|  |  | 5 | 9 498 | 0.89 | 37.57 | 18.01 |
|  |  | 6 | 3 417 | 0.93 | 236.85 | 5.40 |
|  |  |  |  |  |  |  |
| 7-group | -418 983.91 | 1 | 2 145 | 0.93 | 373.91 | 3.36 |
|  |  | 2 | 2 699 | 0.9 | 154.86 | 5.40 |
|  |  | 3 | 6 995 | 0.88 | 52.41 | 12.53 |
|  |  | 4 | 34 461 | 0.77 | 4.49 | 43.24 |
|  |  | 5 | 12 192 | 0.84 | 18.59 | 22.50 |
|  |  | 6 | 4 306 | 0.90 | 97.94 | 8.13 |
|  |  | 7 | 1 832 | 0.93 | 495.35 | 2.80 |

^1^Average Posterior Probabilities; ^2^Odds of Correct Classification; ^3^Posterior Probabilities


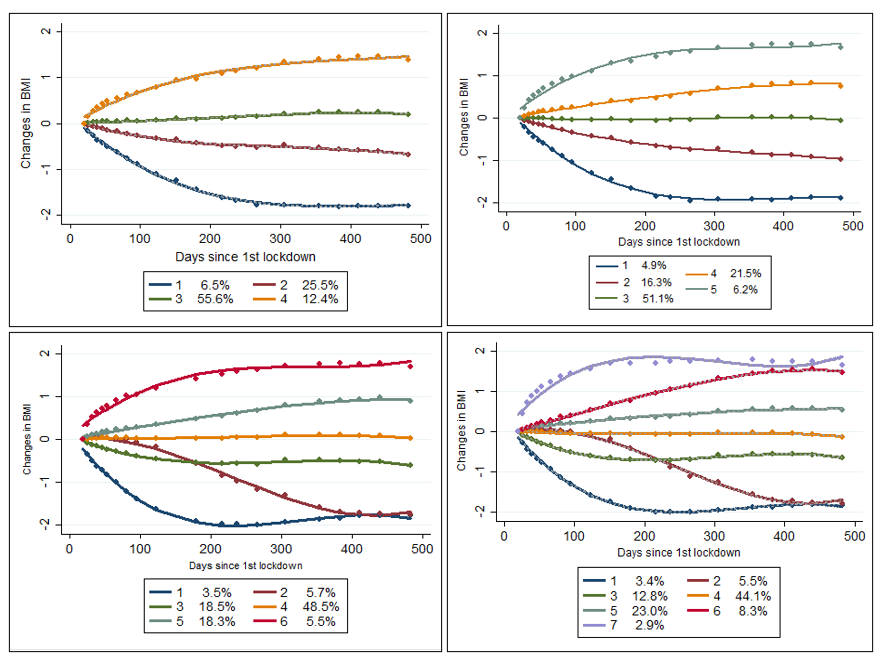
**Figure S1.** Group-based trajectory Analyses. Comparison of 4- to 7-group solutions

**Appendix 2. Sensitivity Analyses: Determinants of changes in BMI (margins based on multinomial regression models)**


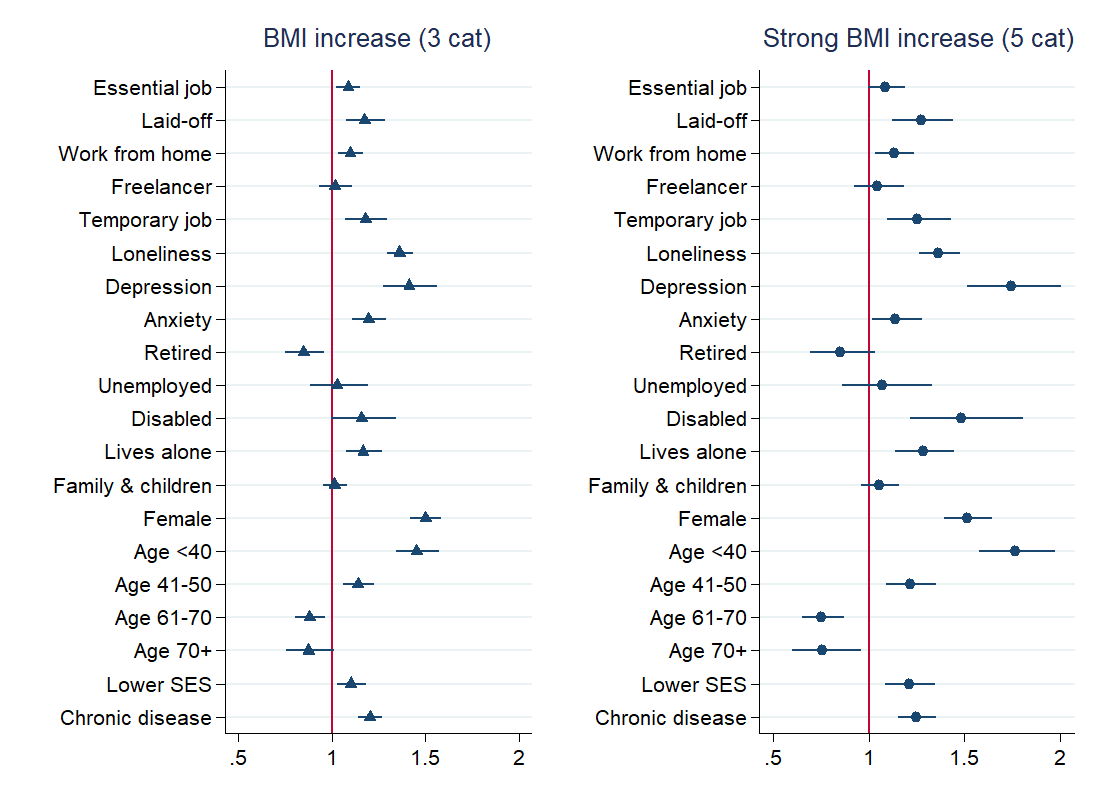


**Figure S2.** Determinants of (strong) BMI increase: comparison between the 3-group and 5-group solution (Odds Ratios)


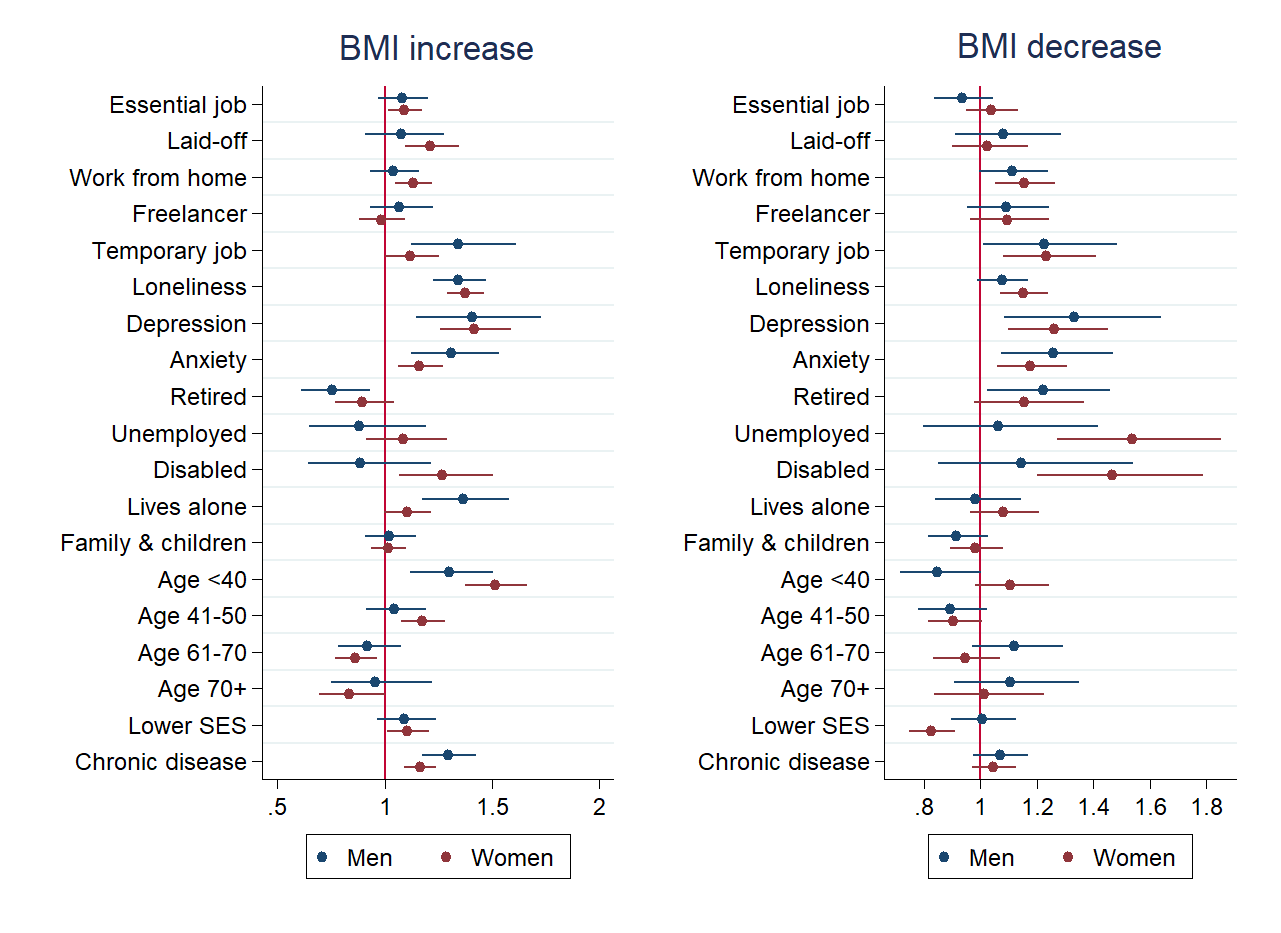


**Figure S3.** Determinants of experiencing BMI increase and decrease, stratified by Gender. Marginal effects based on multinomial regression models (Odds Ratios).

**Table S2. Cumulative effects of work- and health-related events^a^**

| *Ref. Group #2. Stable BMI* | Group #1 BMI Increase | |  | Group #3 BMI Decrease | |
| --- | --- | --- | --- | --- | --- |
|  | OR | CI 95% |  | OR | CI 95% |
| Work-related events (0 events) | 1 |  |  | 1 |  |
| 1 event | 1.19* | 1.10 - 1.30 |  | 1.17* | 1.07 - 1.28 |
| 2 events | 1.26* | 1.15 - 1.38 |  | 1.24* | 1.13 - 1.37 |
| 3 events | 1.37* | 1.21 - 1.57 |  | 1.33* | 1.15 - 1.54 |
| Health-related events (0 events) | 1 |  |  | 1 |  |
| 1 event | 1.39* | 1.32 - 1.47 |  | 1.17* | 1.10 - 1.24 |
| 2 events | 1.74* | 1.59 - 1.90 |  | 1.44* | 1.30 - 1.59 |
| 3 events | 2.19* | 1.96 - 2.46 |  | 1.60* | 1.41 - 1.83 |

^a^Model accounts for the rest of covariates. *p-value <0.01

**Table S3. Cumulative effects all combined events^a^**

| *Ref. Group #2. Stable BMI* | Group #1 BMI Increase | |  | Group #3 BMI Decrease | |
| --- | --- | --- | --- | --- | --- |
|  | OR | CI 95% |  | OR | CI 95% |
| Combined events (0 events) | 1 |  |  | 1 |  |
| 1 event | 1.26* | 1.16 - 1.37 |  | 1.10* | 1.01 - 1.19 |
| 2 events | 1.61* | 1.46 - 1.76 |  | 1.30* | 1.18 - 1.42 |
| 3 events | 1.87* | 1.69 - 2.06 |  | 1.44* | 1.30 - 1.60 |
| 4 events | 2.22* | 1.96 - 2.52 |  | 1.74* | 1.52 - 1.99 |
| 5 events | 2.74* | 2.31 - 3.26 |  | 1.84* | 1.50 - 2.25 |

^a^Model accounts for the rest of covariates. *p-value <0.01

**Appendix 3. Attrition analyses**

The following tables show a set of analyses to assess potential attrition in the study, i.e. whether individuals who were lost at follow-up differed significantly from those who were represented in the whole study. First, Table S4 shows the association between the main variables of interest and the number of observations the participant had. As a complement, Table S5 shows the percentage of participants who matched the inclusion criteria broken down by the main variables of interest.

Both tables show that differences by age are most notable, with younger individuals being underrepresented: while 92% of individuals older than 61 were included in the study, that was only the case for 67% of those under 40. However, differences by other variables of interest are less striking. Regarding BMI at baseline, those with overweight were not more likely to drop out than those with healthy weight. Those with obesity at baseline were slightly less likely to remain in the study, although difference in percentages is rather small (86.6% of those with obesity versus 87.4% of individuals with healthy weight or overweight).

Last, Table S6 shows a regression model with all missing values imputed via multiple imputation chained equations (MICE). The results are practically identical to those with observed values shown in Table 3 in the main document.

**Table S4. Variables associated with the number of responses of the participant. Poisson regression models.**

|  | Nº Observations | |
| --- | --- | --- |
|  | B | S.E. |
| Gender (Male) |  |  |
| Female | 0.05* | 0.01 |
| Age (51-60) |  |  |
| <40 | -0.36* | 0.01 |
| 40-50 | -0.19* | 0.01 |
| 61-70 | 0.21* | 0.01 |
| >70 | 0.25* | 0.01 |
| Household (w. other adults) |  |  |
| Alone | 0.00 | 0.00 |
| With kids | -0.04* | 0.00 |
| Educational Level (High) |  |  |
| Low | -0.09* | 0.01 |
| Middle | -0.06* | 0.01 |
| Employment Status (Employed) |  |  |
| Retired | 0.01* | 0.00 |
| Unemployed | 0.00 | 0.00 |
| Disabled | 0.00 | 0.00 |
| Chronic condition (No) |  |  |
| Yes | 0.04* | 0.01 |
| BMI at baseline (healthy) |  |  |
| Overweight | -0.03* | 0.01 |
| Obesity | -0.04* | 0.01 |

*p-value <0.01

**Table S5. Percentage of participants included in the study^a^ by main variables of interest. ANOVA tests.**

|  | % included | *p* |
| --- | --- | --- |
| Gender |  | *0.23* |
| Male | 83.06% |  |
| Female | 84.29% |  |
| Age group |  | *0.00* |
| < 40 | 66.83% |  |
| 41-50 | 81.45% |  |
| 51-60 | 87.41% |  |
| 61-70 | 92.09% |  |
| >70 | 91.90% |  |
| Household |  | *0.00* |
| With partner/family | 83.02% |  |
| Family with kids | 86.91% |  |
| Alone | 87.06% |  |
| Educational Attainment |  | *0.00* |
| Low | 86.77% |  |
| Middle | 85.57% |  |
| High | 87.31% |  |
| Employment Situation |  | *0.00* |
| Employed | 82.96% |  |
| Retired | 92.75% |  |
| Unemployed | 83.37% |  |
| Disabled | 82.26% |  |
| Other | 76.19% |  |
| Chronic disease |  | *0.00* |
| No | 92.43% |  |
| Yes | 94.05% |  |
| BMI at baseline |  | *0.00* |
| Healthy | 87.40% |  |
| Overweight | 87.41% |  |
| Obese | 86.63% |  |

^a^Inclusion criteria: more than one observation and no missing values in BMI

**Table S6. Multinomial regression models using imputed values.**

| *Ref. Group #2. Stable BMI* | Group #1 BMI Increase | |  | Group #3 BMI Decrease | |
| --- | --- | --- | --- | --- | --- |
|  | OR | CI 95% |  | OR | CI 95% |
| Employment status (Work as usual) | 1 |  |  | 1 |  |
| Retired | 0.89 | 0.80 - 1.00 |  | 1.26* | 1.12 - 1.41 |
| Unemployed | 1.05 | 0.92 - 1.21 |  | 1.42* | 1.23 - 1.64 |
| Disabled | 1.18 | 1.03 - 1.35 |  | 1.43* | 1.23 - 1.49 |
| Work-related events |  |  |  |  |  |
| Essential job | 1.13* | 1.07 - 1.19 |  | 1.05 | 0.99 - 1.12 |
| Laid-off due to COVID | 1.20* | 1.10 - 1.30 |  | 1.09 | 0.99 - 1.20 |
| Work from home | 1.10* | 1.04 - 1.16 |  | 1.14* | 1.07 - 1.22 |
| Freelancer | 1.04 | 0.96 - 1.12 |  | 1.10 | 1.01 - 1.19 |
| Temporary job | 1.19* | 1.09 - 1.30 |  | 1.26* | 1.14 - 1.40 |
| Chronic health condition | 1.20* | 1.14 - 1.27 |  | 1.05 | 0.99 - 1.11 |
| BMI at Baseline ("Healthy weight") | 1 |  |  | 1 |  |
| Overweight | 1.65* | 1.57 - 1.74 |  | 2.28* | 2.15 - 2.42 |
| Obesity | 2.44* | 2.29 - 2.60 |  | 4.36* | 4.07 - 4.67 |
| Health-related events |  |  |  |  |  |
| Loneliness | 1.35* | 1.28 - 1.41 |  | 1.16* | 1.10 - 1.22 |
| Depression | 1.40* | 1.28 - 1.54 |  | 1.29* | 1.16 - 1.43 |
| Anxiety | 1.19* | 1.11 - 1.28 |  | 1.19* | 1.10 - 1.29 |
| Living arrangement (Family, no children) | 1 |  |  | 1 |  |
| Family, with children | 1.07 | 1.01 - 1.14 |  | 0.98 | 0.92 - 1.05 |
| Lives alone | 1.18* | 1.09 - 1.27 |  | 0.97 | 0.89 - 1.05 |
| Age (51-60 years) | 1 |  |  | 1 |  |
| <=40 years | 1.40* | 1.30 - 1.51 |  | 1.05 | 0.96 - 1.14 |
| 41-50 years | 1.12* | 1.05 - 1.19 |  | 0.92 | 0.85 - 0.99 |
| 61-70 years | 0.90 | 0.82 - 0.98 |  | 1.00 | 0.92 - 1.10 |
| >70 years | 0.86 | 0.76 - 0.99 |  | 1.05 | 0.92 - 1.19 |
| Gender (Male) | 1 |  |  | 1 |  |
| Female | 1.49* | 1.42 - 1.57 |  | 1.01 | 0.95 - 1.06 |
| Educational Attainment (High) | 1 |  |  | 1 |  |
| Middle | 1.07 | 1.01 - 1.13 |  | 0.94 | 0.89 - 1.00 |
| Low | 1.11* | 1.04 - 1.18 |  | 0.89* | 0.83 - 0.95 |

*p-value <0.01
